# Comparative complete scheme and booster effectiveness of COVID‐19 vaccines in preventing SARS‐CoV‐2 infections with SARS‐CoV‐2 Omicron (BA.1) and Delta (B.1.617.2) variants: A case–case study based on electronic health records

**DOI:** 10.1111/irv.13121

**Published:** 2023-03-14

**Authors:** Irina Kislaya, André Peralta‐Santos, Vítor Borges, Luís Vieira, Carlos Sousa, Bibiana Ferreira, Ana Pelerito, João Paulo Gomes, Pedro Pinto Leite, Baltazar Nunes, PT COVID‐19 group, Ausenda Machado, Ana Paula Rodrigues, Vasco Ricoca Peixoto, Pedro Casaca, Eugenia Fernandes, Eduardo Rodrigues, Rita Ferreira, Joana Isidro, Miguel Pinto, Sílvia Duarte, Daniela Santos, Luís Meneses, José Pedro Almeida, Ana Matias, Samanta Freire, Teresa Grilo

**Affiliations:** ^1^ Department of Epidemiology National Institute of Health Doutor Ricardo Jorge Lisbon Portugal; ^2^ Public Health Research Centre, NOVA National School of Public Health Universidade NOVA de Lisboa Lisbon Portugal; ^3^ Comprehensive Health Research Centre (CHRC) Universidade NOVA de Lisboa Lisbon Portugal; ^4^ Direção de Serviços de Informação e Análise Direção‐Geral da Saúde Lisbon Portugal; ^5^ Genomic and Bioinformatics Unit, Department of Infectious Diseases National Institute of Health Doutor Ricardo Jorge Lisbon Portugal; ^6^ Departamento de Genética Humana, Instituto Nacional de Saúde Doutor Ricardo Jorge Unidade de Tecnologia e Inovação Lisbon Portugal; ^7^ Unilabs Porto Portugal; ^8^ Algarve Biomedical Center Research Institute (ABC‐RI) Faro Portugal; ^9^ Faculty of Medicine and Biomedical Sciences (FMCB), Campus de Gambelas University of Algarve Faro Portugal; ^10^ Portuguese Red Cross Laboratory Lisbon Portugal

**Keywords:** case–case design, COVID‐19, Delta variant, Omicron variant, SARS‐CoV‐2, vaccine effectiveness

## Abstract

**Background:**

Information on vaccine effectiveness in a context of novel variants of concern (VOC) emergence is of key importance to inform public health policies. This study aimed to estimate a measure of comparative vaccine effectiveness between Omicron (BA.1) and Delta (B.1.617.2 and sub‐lineages) VOC according to vaccination exposure (primary or booster).

**Methods:**

We developed a case–case study using data on RT‐PCR SARS‐CoV‐2‐positive cases notified in Portugal during Weeks 49–51, 2021. To obtain measure of comparative vaccine effectiveness, we compared the odds of vaccination in Omicron cases versus Delta using logistic regression adjusted for age group, sex, region, week of diagnosis, and laboratory of origin.

**Results:**

Higher odds of vaccination were observed in cases infected by Omicron VOC compared with Delta VOC cases for both complete primary vaccination (odds ratio [OR] = 2.1; 95% confidence interval [CI]: 1.8 to 2.4) and booster dose (OR = 5.2; 95% CI: 3.1 to 8.8), equivalent to reduction of vaccine effectiveness from 44.7% and 92.8%, observed against infection with Delta, to −6.0% (95% CI: 29.2% to 12.7%) and 62.7% (95% CI: 35.7% to 77.9%), observed against infection with Omicron, for complete primary vaccination and booster dose, respectively.

**Conclusion:**

Consistent reduction in vaccine‐induced protection against infection with Omicron was observed. Complete primary vaccination may not be protective against SARS‐CoV‐2 infection in regions where Omicron variant is dominant.

## 1 BACKGROUND

The Omicron (BA.1) SARS‐CoV‐2 variant first reported in South Africa on November 24, 2021,^1^ has been designated by the World Health Organization^2^ as a variant of concern (VOC), as it presents several mutations associated with increased transmissibility and higher risk of reinfection.^3^, ^4^ The Omicron (BA.1) variant yields an S‐gene target failure (SGTF) signal (due to the deletion Δ69–70 in the Spike protein) in some polymerase chain reaction (PCR) tests (e.g., TaqPath COVID‐19, ThermoFisher, Waltham, MA, USA), which can be used as a proxy for Omicron detection and differentiation from the circulating Delta (B.1.617.2 and sub‐lineages) variant (in which the Δ69–70 is rarely detected).^5^

The Omicron had a swift rise in Europe becoming dominant in a few weeks in England, Scotland, and Denmark,^6^ and the European Center for Disease Prevention and Control (ECDC) risk assessment^7^ referred that would become dominant in early January 2022 in all European Union (EU) member states. Most EU countries had ongoing mass population vaccination, with considerably high primary vaccination coverage in some countries. Portugal began rolling out the booster campaign, which started in September 2021 for immunosuppressed individuals and older than 50 years old, and in late December 2021, it was expanded to all adults. By December 26, the booster coverage was about 2.5 million doses.^8^

The first studies on neutralization assays revealed an extensive but incomplete escape of Comirnaty BNT162b2‐elicited neutralization,^9^ but booster dose increased the neutralization.^10^ These preliminary in vitro results were confirmed by the first vaccine effectiveness (VE) studies in England and Scotland^11^, ^12^ for symptomatic infections for both complete primary vaccination schemes and booster doses. The UK study estimated a reduction of VE for symptomatic infection with the Omicron with no effect for two ChAdOx1 (AstraZeneca) doses and 8.8% (95% confidence interval [CI]: 7.0 to 10.5) at 25 or more weeks post dose 2 of BNT162b2 (Comirnaty).^11^ Another study in Denmark found similar results with reduced VE against Omicron infection compared with Delta for two doses of BNT162b2 (Comirnaty) and mRNA‐1273 (Moderna), with an increase in VE after a booster dose.^13^ It is still unclear the reasons for differences in the VE of different vaccines and if the decreased VE translate for other countries with different vaccination coverage. It is fundamental to understand how this novel VOC impacts the transmission dynamics in highly vaccinated populations.

To shed some light on those questions, we aim to replicate a study previously performed to estimate the comparative VE of mRNA vaccines between Delta (B.1.617.2 and sub‐lineages) and Alpha (B.1.1.7) VOC.^14^ Our objective is to measure comparative VE (any vaccine) between Omicron and Delta VOC cases according to the complete primary scheme, time since the primary vaccine scheme, and booster dose uptake. Additionally, we intend to translate comparative VE estimates between Omicron versus Delta VOC into VE estimates against Omicron infection using published estimates of COVID‐19 VE against Delta.

## 2 METHODS

### 2.1 Study design

To estimate a measure of comparative COVID‐19 VE of a complete primary vaccination scheme and of the booster dose against the SARS‐CoV‐2 Omicron (BA.1) versus Delta (B.1.617.2 and sub‐lineages) VOC infections, we used a case–case study design.^15^ A similar approach has been used in Portugal to compare COVID‐19 VE against Alpha and Delta VOC and has been previously described elsewhere.^14^ This design has been proven useful to address the questions of COVID‐19 VE in the context of novel VOC emergence comparing directly the odds of vaccination between RT‐PCR‐positive cases infected with different VOC. Considering cases of the Omicron SARS‐CoV‐2 infection as cases of interest and Delta infections as the reference group, higher vaccination odds in Omicron cases in a case–case study are indicative of lower effectiveness of COVID‐19 vaccines against the Omicron compared with the Delta VOC (supporting information).

The study period covered 3 weeks (December 6–26, 2021), starting on Week 49 with a predominant circulation of the Delta VOC and relative frequency of the Omicron of 4.2%^16^ until Week 51, when the Omicron became predominant (50.8%).^17^

The target population included individuals resident in mainland Portugal aged 12 or more years old (eligible for vaccination at the time of the data collection)^18^ with positive RT‐PCR notified to the mandatory National Epidemiological Surveillance Information System (SINAVE) during the study period. To evaluate the effect of primary vaccination, data were restricted to individuals without a history of previous SARS‐CoV‐2 infection or vaccine booster. To access the effect of the booster dose, we restricted the sample to those aged 50 or more years old because younger age groups were not yet eligible for the booster vaccination at the time of the study and to those without a history of previous SARS‐CoV‐2 infection.^18^ We excluded individuals with missing data on National Health Service User number, age, sex, place of residence, or diagnosis date from all analyses.

### 2.2 Data sources

#### 2.2.1 SARS‐CoV‐2 cases

We linked laboratory data on Ct values and whole‐genome sequencing (WGS) on SARS‐CoV‐2‐positive cases collected by the National SARS‐CoV‐2 Genomic Surveillance Network and three private molecular biology laboratories (UNILABS, Algarve Biomedical Center, and Portuguese Red Cross) to the national electronic vaccination register (VACINAS) and the National Epidemiological Surveillance Information System that contains basic demographic information on cases and data of previous SARS‐CoV‐2 infections since the beginning of the pandemic. Deterministic data linkage was performed on January 4, 2022, by the General Directorate of Health using the National Health Service User number that uniquely identifies individuals in all national administrative health registries. Based on the National Health Service User number, records for duplicated samples, collected within 90 days, were removed maintaining only data from the first collected sample registry for each SARS‐CoV‐2 infection.

#### 2.2.2 Variant classification

As an outcome, we considered laboratory‐confirmed SARS‐CoV‐2 infection (either symptomatic or asymptomatic). SARS‐CoV‐2 variants were classified by viral WGS or inferred for non‐sequenced samples based on spike (S) gene target failure (SGTF) using the TaqPath™ Covid 19 CE IVD RT‐PCR Kit (Thermo Scientific™) assay, as follows: no S‐gene amplification (SGTF = Omicron BA.1) and S‐gene amplification (non‐SGTF = Delta). TaqPath S‐positive samples could be confidently classified as Delta because this VOC was dominant in Portugal since the last summer (weekly frequencies above 99% between Weeks 30 and 47, when Omicron started emerging) (https://insaflu.insa.pt/covid19/).

#### 2.2.3 Vaccination status

In Portugal, four vaccines were authorized for primary vaccination at the time of the study: three brands (BNT162b2 [Comirnaty, https://www.pfizer.com] or mRNA‐1273 SARS‐CoV‐2 [Moderna, https://www.modernatx.com] and ChAdOx1 nCoV‐19 [AstraZeneca, https://www.astrazeneca.com] with a two‐dose regimen) and one brand (Ad26.COV2‐S [Janssen, https://www.janssen.com] with a single‐dose regimen). mRNA vaccines (Comirnaty and Moderna) were used for the boost.^18^ Both homologous and heterologous vaccination schemes were considered.

Vaccination exposure, obtained through the electronic nationwide register VACINAS, was classified as (i) unvaccinated (no register of vaccine administration); (ii) partial primary vaccination (SARS‐CoV‐2 infection diagnosis less than 14 days after completing the primary vaccination scheme according to the product used); (iii) complete primary vaccination (SARS‐CoV‐2 infection diagnosis 14 or more days following the complete vaccination scheme according to the product characteristics: 14 days or more days after the second dose of mRNA or Vaxzevria vaccines uptake and 14 days after the single dose of the Janssen COVID‐19 vaccine uptake); (iv) partial boost (SARS‐CoV‐2 infection diagnosis less than 14 days after booster dose uptake); and (v) boost complete (SARS‐CoV‐2 infection diagnosis 14 or more days following booster dose uptake).

To account for time since vaccination uptake, we additionally considered three categories within the complete primary vaccination: (i) complete primary vaccination less than 113 days (16 weeks); (ii) complete primary vaccination 113–168 days (17–24 weeks); and (iii) complete primary vaccination more than 168 days (25 or more weeks).

To avoid small sample size bias, we will not present estimates for vaccine exposure categories with sample size *n* < 20.

### 2.3 Ethical statement

The genomic surveillance of SARS‐CoV‐2 in Portugal is regulated by the Assistant Secretary of State and Health Executive Order (Despacho No. 331/2021) of January 11, 2021. The research on genomic epidemiology of novel coronavirus (SARS‐CoV‐2) received the clearance of the Ethics Committee of Instituto Nacional de Saúde Doutor Ricardo Jorge on March 30, 2021. As this study is based on electronic health records data linkage, the requirement for patients' informed consent was waived by the Ethics Committee.

### 2.4 Statistical analysis

Characteristics of participants infected with Omicron and Delta VOC were compared using the chi‐square test. Logistic regression adjusted for age group, sex, region of residence, week of diagnosis, and laboratory of origin was used to estimate adjusted odds of complete/boosted vaccination in Omicron‐infected cases compared with Delta‐infected SARS‐CoV‐2 cases. If the odds of vaccination between Omicron and Delta cases are similar, we expect to obtain an odds ratio (OR) = 1, a proxy of no difference in VE. If the odds of vaccination among Omicron cases are higher compared with Delta, we expect an OR to be greater than one (OR > 1) and as such lower VE against Omicron compared with Delta VOC. In contrast, an OR smaller than one (OR < 1) will indicate higher VE against Omicron in comparison with Delta VOC.

We also provide estimates of VE against the Omicron laboratory‐confirmed infection (either symptomatic or asymptomatic) for the complete primary vaccination scheme and the booster dose by combining previously published VE estimates against Delta and OR estimated in this study using the following formula:

$${\mathrm{VE}}_{\text{Omicron}}=1-\left[\left(1-{\mathrm{VE}}_{\text{Delta}}\right)\cdot {\text{OR}}_{\text{case}-\text{case}}\right]$$

where ${\mathrm{VE}}_{\text{Omicron}}$ represents COVID‐19 VE against Omicron VOC, ${\mathrm{VE}}_{\text{Delta}}$ represents COVID‐19 VE estimates against Delta VOC, and ${\text{OR}}_{\text{case}-\text{case}}$ represents the ratio of vaccination odds between Omicron cases versus Delta cases obtained through a case–case design.

To account for uncertainty from the CIs around point estimates, we used Monte Carlo simulations, considering that the logarithm of ${\text{OR}}_{\text{case}-\text{case}}$ and the logarithm of ($1-{\mathrm{VE}}_{\text{Delta}}$) are normally distributed. A more detailed description of Monte Carlo simulations is provided in the supporting information.

### 2.5 Sensitivity analysis

To assess the bias of misclassification error associated with the SGTF method, we included only cases identified exclusively through WGS. We also restricted the analysis to the samples with Ct values below 25 (Ct < 25) as samples with higher Ct values are less likely to be successfully sequenced by WGS.

To account for the effect of previous infection, we performed an additional analysis including cases with previous infection. For this, we considered the following levels of exposure, combining information on vaccination status and previous infection: (i) unvaccinated without previous infection; (ii) unvaccinated with previous infection; (iii) partially or completely vaccinated with previous infection; (iv) completely vaccinated without previous infection; and (v) booster vaccination without previous infection. Previous infection was defined as having laboratory confirmation of SARS‐CoV‐2 at least 90 days before current diagnosis by RT‐PCR or rapid antigen test.

## 3 RESULTS

**FIGURE 1:**
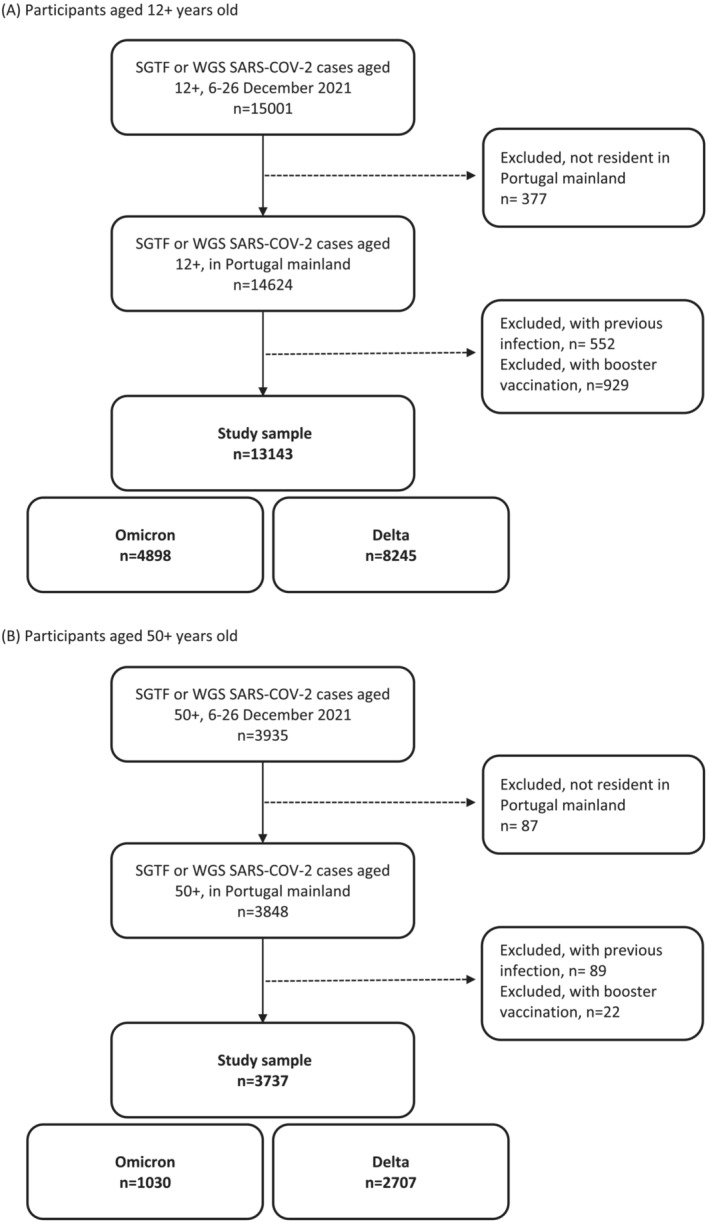
Participants selection flowchart, Portugal, Weeks 49–51, 2021. (A) Participants aged 12+ years old. (B) Participants aged 50+ years old. SGTF, S‐gene target failure; WGS, whole‐genome sequencing.

Of a total of 15,001 samples collected during the study period for the population aged 12 or more years old, 13,143 were included in the main analysis (Figure 1). Of those 4898 (37.3%) were classified as Omicron and 8245 (62.7%) as Delta. The distribution of Omicron cases differed from the distribution of Delta cases by all considered covariates (Table 1).

**TABLE 1:** Distribution of SARS‐CoV‐2 Omicron and Delta cases by method of classification, sex, age, region, week of diagnosis, and COVID‐19 vaccination status.

	Omicron		Delta		*p*‐value^a^
	*n*	%	*n*	%	
**Method of VOC classification**					
S‐gene	4732	96.6	7705	93.5	
WGS	138	2.8	452	5.5	
WGS + S‐gene	28	0.6	88	1.1	
**Sex**					0.009
Female	2535	51.76	4074	49.41	
Male	2363	48.24	4171	50.59	
**Age group**					<0.001
12–19	671	13.7	648	7.9	
20–29	1508	30.8	1625	19.7	
30–39	967	19.7	1779	21.6	
40–49	961	19.6	1913	23.2	
50–64	745	15.2	1825	22.1	
65+	46	0.9	455	5.5	
**Region**					<0.001
Alentejo	258	5.3	431	5.2	
Algarve	50	1.0	153	1.9	
Centro	414	8.5	975	11.8	
Norte	2592	52.9	5307	64.4	
Lisboa	1584	32.3	1379	16.7	
**Week**					<0.001
49	184	3.8	3428	41.6	
50	1032	21.1	2960	35.9	
51	3682	75.2	1857	22.5	
**Vaccination brand**					<0.001
Unvaccinated	315	6.4	888	10.8	
AstraZeneca	274	5.6	717	8.7	
Janssen	876	17.9	1524	18.5	
Moderna	639	13.1	696	8.4	
Comirnaty	2794	57.0	4420	53.6	

Abbreviations: VOC, variant of concern; WGS, whole‐genome sequencing.

^a^ *p*‐value of chi‐square test.

### 3.1 Main results

We observed higher odds of complete vaccination in Omicron cases versus Delta (OR = 2.1), suggesting reduced effectiveness of complete vaccination schemes with mRNA or viral vector vaccines in preventing SARS‐CoV‐2 infection with the novel Omicron (BA.1) VOC in the Portuguese population aged 12 or more years old (Table 2).

**TABLE 2:** Crude and adjusted odds ratios of vaccine infection breakthrough in Omicron (BA.1) cases compared with Delta (B.1.617.2) SARS‐CoV‐2 cases, Portugal, Weeks 49–51, 2021.

Vaccination status	Omicron	Delta	Crude odds ratio (95% CI)	Confounding‐adjusted^a^ odds ratio (95% CI)
	*n* (%)	*n* (%)		
**Population aged 12 or more years old, *n* = 13,143**	4898 (37.3)	8245 (62.7)		
Unvaccinated	315 (6.4)	888 (10.8)	ref	ref
Partial primary vaccination	68 (1.4)	112 (1.4)	1.7 (1.2 to 2.4)	1.5 (0.97 to 2.2)
Complete primary vaccination	4515 (92.2)	7245 (87.8)	1.8 (1.5 to 2.0)	2.1 (1.8 to 2.4)
Complete primary vaccination < 113 days	1002 (22.2)	1141 (15.5)	2.5 (2.1 to 2.9)	2.3 (1.9 to 2.8)
Complete primary vaccination 113–168 days	2640 (58.5)	4313 (59.5)	1.7 (1.5 to 2.0)	2.0 (1.7 to 2.4)
Complete primary vaccination 169+ days	873 (19.3)	1791 (24.7)	1.4 (1.2 to 1.6)	1.9 (1.6 to 2.3)
**Cases with Ct < 25, *n* = 11,235**				
Unvaccinated	261 (6.1)	697 (10.1)	ref	ref
Partial primary vaccination	57 (1.3)	83 (1.2)	1.8 (1.3 to 2.6)	1.7 (1.1 to 2.7)
Complete primary vaccination	3979 (92.6)	6158 (88.8)	1.7 (1.5 to 2.0)	2.1 (1.8 to 2.6)
**Cases classified by WGS, *n* = 706**				
Unvaccinated	20 (12.1)	110 (20.4)	ref	ref
Partial primary vaccination	5 (3.0)	7 (1.3)	NE	NE
Complete primary vaccination	141 (84.9)	423 (78.3)	1.8 (1.1 to 3.1)	2.7 (1.4 to 5.0)
**Accounting for previous infection, *n* = 13,647**				
Unvaccinated without previous infection	315 (6.0)	888 (10.6)	ref	ref
Unvaccinated with previous infection	327 (6.2)	108 (1.3)	8.5 (6.6 to 11.0)	9.0 (6.6 to 12.3)
Partial without previous infection	68 (1.3)	112 (1.3)	1.7 (1.2 to 2.4)	1.5 (0.97 to 2.2)
Partial/complete with previous infection	44 (0.84)	25 (0.3)	5.0 (3.0 to 8.2)	5.1 (2.6 to 9.9)
Complete without previous infection	4515 (85.7)	7245 (88.6)	1.8 (1.5 to 2.0)	2.1 (1.8 to 2.4)

Abbreviations: CI, confidence interval; NE, not estimated due to small sample size; ref, reference; WGS, whole‐genome sequencing.

^a^ Logistic regression model adjusted for sex, age group, region, week of diagnosis, and laboratory of origin; SARS‐CoV‐2 infection was considered as an outcome, with reference category being infected with Delta variant of concern.

Considering time since complete scheme vaccine uptake, we found statistically significant higher odds of vaccination in Omicron cases for all time intervals since complete vaccination, indicative of VE reduction regardless of time since complete vaccination.

We estimated higher odds of reinfection with Omicron, regardless of vaccination status (Table 2), suggesting a lower level of protection conferred by previous infection as well as by hybrid immunity (previous infection + vaccination) against Omicron compared with Delta.

Sensitivity analysis restricted to cases classified only by WGS for the population aged 12 years or older corroborated the observed differences in odds of complete vaccination between the Omicron and Delta cases, leading to a slightly higher point estimate of OR (OR = 2.7). Restriction to samples with Ct < 25 also resulted in no relevant change in vaccine breakthrough OR estimates for the population aged 12 years or older.

Our analysis regarding the comparative booster dose VE against Omicron versus Delta cases was restricted to 3737 cases collected for the population aged 50 or more years old without previous SARS‐CoV‐2 infection (Table 3). Cases' characteristics by vaccination status are shown in the supporting information.

**TABLE 3:** Crude and adjusted odds ratios of booster dose vaccine infection breakthrough in Omicron (BA.1) cases compared with Delta (B.1.617.2) SARS‐CoV‐2 cases, Portugal, individuals aged 50 or more years of age, Weeks 49–51, 2021.

Vaccination status	Omicron *n*	Delta *n*	Crude odds ratio (95% CI)	Confounding‐adjusted^a^ odds ratio (95% CI)
**Population aged 50 or more years old, *n* = 3737**				
Unvaccinated	38	176	ref	ref
Partial primary vaccination	7	15	NE	NE
Complete primary vaccination	746	2089	1.7 (1.2 to 2.4)	1.7 (1.1 to 2.7)
Booster less than 14 days	97	283	1.6 (1.1 to 2.4)	2.1 (1.3 to 3.6)
Booster complete (14+ days)	149	159	4.3 (2.9 to 6.6)	5.2 (3.1 to 8.8)
**Samples with Ct < 25, *n* = 3091**				
Unvaccinated	34	114	ref	ref
Partial primary vaccination	7	15	NE	NE
Complete primary vaccination	655	1528	1.3 (0.9 to 1.9)	1.4 (0.9 to 2.2)
Booster less than 14 days	89	224	1.3 (0.8 to 2.1)	1.9 (1.1 to 3.3)
Booster complete (14+ days)	122	125	3.3 (2.1 to 5.2)	4.3 (2.4 to 7.6)

Abbreviations: CI, confidence interval; NE, not estimated due to small sample size; ref, reference.

^a^ Logistic regression model adjusted for sex, age group, region, week of diagnosis, and laboratory of origin; SARS‐CoV‐2 infection was considered as an outcome, with reference category being infected with Delta variant of concern.

We observed higher odds of booster vaccination in Omicron cases (OR = 5.2; 95% CI: 3.1 to 8.8) compared with Delta (Table 3). This relative reduction in the protection conferred by the booster dose was more pronounced compared with the one observed for complete vaccination in this age group (OR = 1.7; 95% CI: 1.1 to 2.6).

In sensitivity analysis, restriction to cases with Ct < 25 led to a reduction in OR estimates for both complete primary vaccination (OR = 1.4; 95% CI: 0.9 to 2.2) scheme and booster vaccination (OR = 4.3; 95% CI: 2.4 to 7.6).

Due to the small sample size of cases classified by WGS and the absence of cases with previous infection among those with booster doses, it was not possible to perform any other sensitivity analysis for the population aged 50 or more years old.

### 3.2 VE against Omicron (BA.1) VOC

Using previously published data on VE against symptomatic Delta infection (referred to September–December 2021),^19^ we estimated VE against infection with the Omicron VOC of 33.5% (95% CI: 18.9% to 45.2%) for the complete primary vaccination scheme with Comirnaty and of 63.1% (95% CI: 37.0% to 78.3%) for the booster with Comirnaty (Table 4).

**TABLE 4:** Estimates of complete primary scheme COVID‐19 Comirnaty and Vaxzevria vaccine effectiveness and Comirnaty booster dose vaccine effectiveness against Omicron VOC, based on the combination of vaccine effectiveness estimates against Delta VOC previously published^19^ and vaccine breakthrough odds ratio infection in Omicron versus Delta VOC cases.

	Vaccine effectiveness against Delta VOC^19^	Odds ratio of vaccine breakthrough infection in Omicron cases versus Delta cases	Vaccine effectiveness against Omicron VOC
**Comirnaty vaccine**			
Complete vaccine scheme	65.3 (64.7 to 65.9)	2.1 (1.8 to 2.4)	33.5% (18.9% to 45.2%)
Booster dose of Comirnaty, 35–69 days	92.9 (92.5 to 93.3)	5.2 (3.1 to 8.8)	63.1% (37.0% to 78.3%)
**Vaxzevria vaccine**			
Complete vaccine scheme	44.7 (43.7 to 45.6)	2.1 (1.8 to 2.4)	−6.0% (−29.2% to 12.7%)
Booster dose of Comirnaty, 35–69 days	92.8 (92.2 to 93.4)	5.2 (3.1 to 8.8)	62.7% (−35.7% to 77.9%)

Abbreviation: VOC, variant of concern.

For those vaccinated with the Vaxzevria, we obtained VE estimates of −6.0% (95% CI: −29.2% to 12.7%) for the complete primary vaccination and 62.7% (95% CI: 35.7% to 77.9%) for ≥10 weeks following the subsequent booster with Comirnaty vaccine.

## 4 DISCUSSION

For the Portuguese population aged 12 or more years old, our results show higher odds of complete primary vaccination in cases infected by Omicron (BA.1) VOC compared with Delta (B.1.617.2 and sub‐lineages) VOC cases (OR = 2.1). This pattern was observed for all time intervals since the completion of the primary vaccination scheme according to the product characteristics (OR = 2.3 for complete vaccination less than 113 days [16 weeks], OR = 2.0 for 113–168 days since complete vaccination, and OR = 1.9 for more than 169 days [25 weeks] since complete vaccination). This finding on the consistent difference between Omicron and Delta regardless of time since vaccination was similar to primary VE reduction against Omicron VOC observed in the UK^11^ and the Netherlands.^20^ Translation of case–case OR estimates to VE estimates against Omicron VOC infection resulted in substantially lower and even null COVID‐19 vaccine effect for complete primary vaccination against Omicron (VE ranged between −6.0% and 33.5%). These results are indicative of low or null protection of complete primary vaccination against SARS‐CoV‐2 infection also observed in other countries.^11^, ^12^

For the booster dose, available at the time of the study for the population aged 50 or more years old, we estimated an OR of 5.2 (95% CI: 3.1 to 8.8) that also suggested a marked reduction of protection against infection (regardless of presence of symptoms) with Omicron compared with Delta. The measure of comparative VE estimated for the booster dose was even higher than for the complete primary vaccination scheme. The interpretation of relative vaccine effects is not straightforward. The magnitude of the reduction of vaccine‐induced protection depends on the baseline value of the VE against Delta VOC. Because the booster has been shown to be more effective against Delta VOC infection^11^ than the primary vaccination, there was more potential for reduction of vaccine‐induced protection with the booster and, as such, the measure of comparative VE estimated in our study was higher for the booster dose. The translation of case–case OR estimates in VE of the booster dose against Omicron VOC infection led to estimates ranging from 62.7% to 63.1%. These results were consistent with previous findings from a UK study that reported moderate VE of the booster dose against symptomatic infections with Omicron up to 10 weeks after the booster uptake.^11^ However, the protection induced by booster dose may be short‐lived, because considerable VE waning, in particular against SARS‐CoV‐2 infection, has been previously reported in the literature for primary vaccination.^21^ So further studies are required to monitor booster VE against Omicron infection for a long time.

Our results regarding the protection afforded by documented previous infection in unvaccinated populations also suggest reduced protection against Omicron compared with Delta VOC, which is consistent with an increased number of reinfections reported to the national surveillance system SINAVE following Omicron emergence. Similar results regarding the previous infection were also observed in the Netherlands.^20^ Omicron BA.1 has accumulated more mutations than Delta; hence, even a previous infection might not account for significant protection for this VOC.

Our study also suggests a significant reduction in the protection conferred by hybrid immunity (OR = 5.1) against infection with Omicron compared with Delta. Previous literature identified a hybrid immunity, resulting from vaccination and previous infection, as the most effective to protect against severe forms of the disease^22^; however, for SARS‐CoV‐2 infection with Omicron VOC, the hybrid immunity might not be so protective, as also highlighted in the recent studies from Qatar.^23^

Our study has several limitations. First, we were unable to differentiate between symptomatic and asymptomatic infections. Second, our study was based on RT‐PCR tests, so rapid antigen tests widely used in Portugal for SARS‐CoV‐2 diagnosis^24^ were not covered by our data. However, the eligibility for RT‐PCR tests and rapid antigen tests did not change over the study period. Although this study included cases samples from National SARS‐CoV‐2 Genomic Surveillance Network (which performs nationwide random weekly sampling for WGS)^18^ and three major clinical pathology laboratories (which provide broad RT‐PCR testing services to the community), we cannot exclude that cases included in our study might not be representative of the overall infections detected in Portugal during Weeks 49–51, 2021. Hence, we compared the age group, sex, and region of the study sample and the overall cases notified with infection in the same period. We found no significant differences in the sex and age group distribution between the universe of identified cases through rapid or RT‐PCR testing and notified SINAVE and the study sample. However, the North region tends to be overrepresented in our study sample. Third, incentives for testing changed during the study period; negative tests regardless of vaccination status became required to go to restaurants and hotels and to participate in cultural events.^25^ This change may affect study results. However, the selection of cases for the study sample was independent of the VOC type and vaccination status. Fourth, this study did not collect data on comorbidities or considered relevant confounding variables in VE research. So, we were not able to adjust for comorbidities in our models, nor estimate the proportion of those with comorbidities by vaccination status. This can affect study results because individuals with comorbidities, such as immunosuppressed patients, were prioritized for additional vaccination doses in Portugal within the primary vaccination regimen.^18^ Also, due to the small proportion of vaccinated with AstraZeneca and Janssen in the study sample, we were not able to estimate comparative VE by vaccine brand. Finally, to translate the case–case result to VE estimates, we used previously published estimates of VE against symptomatic infection with Delta, whereas our study sample included notified infections regardless of their symptoms. In addition, Delta VE data used to extrapolate VE for Omicron were from an earlier period,^19^ with a shorter time since vaccination. Waning of protection conferred by the vaccine may mean that VE against Omicron from a more recent time, with a longer time since vaccination, may be overestimated.

Among study strengths, we should mention its large sample size due to the use of surveillance data, well‐established and high positive predictive value in the SGTF method to classify infections as Delta or Omicron variants, and the robustness of results when we changed the sampling strategy in our sensitivity analyses (WSG‐only or restriction to Ct < 25).

Although direct comparisons to other studies are challenging due to differences in methodology, outcome and exposure definitions, vaccination calendar, eligibility for boosters, vaccine brand‐specific policies, testing patterns, and other non‐pharmacological interventions in place, our findings corroborate the general trend. More specifically, our results support a marked reduction of primary and booster vaccination schemes' effectiveness in preventing Omicron infections compared with Delta observed in other countries.^11^, ^12^ Our findings suggest that complete primary vaccination may not be protective against SARS‐CoV‐2 infection in regions where the Omicron variant is dominant.

## AUTHOR CONTRIBUTIONS

**Irina Kislaya:** Conceptualization; formal analysis; methodology; writing—original draft. **André Peralta‐Santos:** Investigation; writing—original draft. **Vítor Borges:** Investigation; writing—review and editing. **João Paulo Gomes:** Resources; writing—review and editing. **Carlos Sousa:** Investigation; resources; writing—review and editing. **Pedro Pinto Leite:** Conceptualization; writing—review and editing. **Luís Vieira:** Investigation; resources; writing—review and editing. **Bibiana Ferreira:** Investigation; resources; writing—review and editing. **Ana Pelerito:** Investigation; resources; writing—review and editing. **Baltazar Nunes:** Conceptualization; methodology; project administration; writing—review and editing. **Members of PT COVID‐19 group:** Investigation; writing—review and editing.

## CONFLICT OF INTEREST STATEMENT

Dr. Peralta‐Santos reports to participate as a speaker in scientific meetings sponsored by Pfizer. Other authors report no potential conflicts of interest.

## ETHICS APPROVAL STATEMENT

The genomic surveillance of SARS‐CoV‐2 in Portugal is regulated by Assistant Secretary of State and Health Executive Order (Despacho No. 331/2021) of January 11, 2021. The research on genomic epidemiology of novel coronavirus (SARS‐CoV‐2) received the clearance of the Ethics Committee of Instituto Nacional de Saúde Doutor Ricardo Jorge on March 30, 2021. As this study is based on electronic health records data linkage, the requirement for patients' informed consent was waived by the Ethics Committee.

## Supporting information


**Table S1.** Classification of cases by vaccination, positivity status and SARS‐CoV‐2 variant.
**Table S2.** Distribution of SARS‐CoV‐2 Omicron (BA.1) and Delta (B1.617.2) cases by method of classification, sex, age, region, week of diagnosis and COVID‐19 vaccination status for 50 or more years old.

## Data Availability

The data that support the findings of the study were made available under a license that the author does not have permission to share. Requests to access the raw data should be directed to the data owner, the General Directorate of Health.
