# Supplementary material for: Comparative complete scheme and booster effectiveness of COVID‐19 vaccines in preventing SARS‐CoV‐2 infections with SARS‐CoV‐2 Omicron (BA.1) and Delta (B.1.617.2) variants: A case–case study based on electronic health records
Source: Influenza Other Respir Viruses. 2023 Mar 14;17(3):e13121. doi: 10.1111/irv.13121 (PMC10014519; doi:10.1111/irv.13121)
Supplement: Supplementary file 1 — Table S1. Classification of cases by vaccination, positivity status and SARS‐CoV‐2 variant. Table S2. Distribution of SARS‐CoV‐2 Omicron (BA.1) and Delta (B1.617.2) cases by method of classification, sex, age, region, week of diagnosis and COVID‐19 vaccination status for 50 or more years old. [file IRV-17-e13121-s001.docx]

**Supplementary data**

**Estimation of comparative vaccine effectiveness in case-case study design**

The most common approach to estimate the vaccine effectiveness (VE) for COVID-19 vaccines is the test-negative case-control (TND) design. In this study design the VE is estimated by the odds ratio of vaccination between SARS-CoV-2 positive (cases) patients versus SARS-CoV-2 negative (controls) patients (VE=(1-OR)*100%).

Variant-specific vaccine effectiveness estimates using the TND study design can be obtained by restricting the TND to cases positive to specific variant versus negative controls (Table S1)

Table S1 — Classification of cases by vaccination, positivity status and SARS-CoV-2 variant

|  | Omicron+ | Delta+ | Negative controls |
| --- | --- | --- | --- |
| Vaccinated | a | k | b |
| Unvaccinated | c | m | d |

Considering this and assuming for simplicity absence of confounding bias, the variant specific vaccine effectiveness could be estimated by:

$VE_{omicron}=\left( 1-OR_{omicron} \right)\times100\%$ and $VE_{delta}=\left( 1-OR_{delta} \right)\times100\%$

where $OR_{omicron}$ and $OR_{delta}$ are obtained as follows:

$OR_{omicron}=\frac{\left( a/c \right)}{\left( b/d \right)}$ and $OR_{delta}=\frac{\left( k/m \right)}{\left( b/d \right)}$

Considering that $OR_{omicron}$and $OR_{delta}$ are measures of the effect of the COVID-19 vaccine against respectively the Omicron and the Delta variant, the ratio of these two OR’s is an estimate of the comparative effect of the vaccine against the Omicron vs the Delta variant. And we could even consider it a measure of the relative effectiveness of the COVID-19 vaccine (rVE) against the omicron vs the delta variant.

$$rVE_{omicron/delta}=\frac{OR_{omicron}}{OR_{delta}}=\frac{\frac{\left( a/c \right)}{\left( b/d \right)}}{\frac{\left( k/m \right)}{\left( b/d \right)}}=\frac{\left( a/c \right)}{\left( k/m \right)}=OR_{case-case}$$

This result shows that the rVE against Omicron vs Delta variant is equal to the OR obtained in case-case study where the odds of vaccination is compared between cases of omicron variant versus cases of the delta variant.

Table S2. Distribution of SARS-CoV-2 Omicron (BA.1) and Delta (B1.617.2) cases by method of classification, sex, age, region, week of diagnosis and COVID-19 vaccination status for 50 or more years old

|  | Omicron | | Delta | |  |
| --- | --- | --- | --- | --- | --- |
|  | n | % | n | % | p-value |
| Sample type |  |  |  |  | <0.001 |
| S-gene | 998 | 96.9 | 2431 | 89.8 |  |
| WGS | 28 | 2.7 | 243 | 9.0 |  |
| WGS+S-gene | 4 | 0.4 | 33 | 1.2 |  |
| Sex |  |  |  |  | 0.293 |
| Female | 522 | 50.7 | 1424 | 52.6 |  |
| Male | 508 | 49.3 | 1283 | 47.4 |  |
| Age group |  |  |  |  | <0.001 |
| 50-64 | 837 | 81.3 | 1945 | 71.9 |  |
| 65+ | 193 | 18.7 | 762 | 28.2 |  |
| Region |  |  |  |  | <0.001 |
| Alentejo | 73 | 7.1 | 162 | 6.0 |  |
| Algarve | 11 | 1.1 | 65 | 2.4 |  |
| Centro | 90 | 8.7 | 367 | 13.6 |  |
| Norte | 519 | 50.4 | 1756 | 64.9 |  |
| AM Lisboa | 337 | 32.7 | 357 | 13.2 |  |
| Week |  |  |  |  | <0.001 |
| 49 | 45 | 4.4 | 1178 | 43.5 |  |
| 50 | 190 | 18.5 | 949 | 35.1 |  |
| 51 | 795 | 77.2 | 580 | 21.4 |  |
| Vaccination Status |  |  |  |  | <0.001 |
| Unvaccinated | 38 | 3.7 | 176 | 6.5 |  |
| Vaxzevria | 197 | 19.1 | 581 | 21.5 |  |
| Janssen | 90 | 8.7 | 282 | 10.4 |  |
| Spikevax | 117 | 11.4 | 236 | 8.7 |  |
| Comirnaty | 588 | 57.1 | 1432 | 52.9 |  |

**Construction of empirical distributions and estimation of confidence intervals for COVID-19 vaccine effectiveness (VE) against Omicron using data on VE against Delta and odds ratio from case-case study design**

We computed estimates of vaccine effectiveness against the Omicron for complete primary vaccination scheme and for the booster dose by combining previously published vaccine effectiveness estimates against Delta and OR estimated in this study using the following formula:

$${VE}_{Omicron}=1-\left[ \left( 1-{VE}_{Delta} \right)\cdot{OR}_{case-case} \right]$$

where ${VE}_{Omicron}$ represents COVID-19 vaccine effectiveness against Omicron VOC, ${VE}_{Delta}$ COVID-19 vaccine effectiveness estimates against Delta VOC and ${OR}_{case-case}$ the ratio of vaccination odds between Omicron cases versus Delta cases obtain through a case-case design.

To estimate 95% confidence intervals for COVID-19 VE against Omicron we used Monte Carlo simulations. First, we constructed empirical distributions for all input parameters, namely of vaccine effectiveness against Delta ${(VE}_{Delta})$ and ${OR}_{case-case}$ from case-case design.

To construct empirical distribution of ${VE}_{Delta}$ we assumed a normal distribution for log(1-${VE}_{Delta}$), since COVID-19 ${VE}_{Delta}$ estimates were obtained in test-negative design study as ${VE}_{Delta}$=1-Odds ratio${(OR}_{Delta}$), estimated by logistic regression model, as ${OR}_{Delta}$=exp($\beta_{ve\_delta}$), where $\beta_{ve\_delta}$ represents coefficient from logistic regression model for vaccination exposure. We transformed ${VE}_{Delta}$ into $\hat{\beta_{ve\_delta}}=log(1-\hat{{VE}_{Delta})}$ and ${VE}_{Delta}$ 95% confidence interval upper $\hat{{VE}_{Delta}(u)}$ and lower $\hat{{VE}_{Delta}(l)}$ bounds into $\hat{\beta_{ve\_delta(l)}}=log(1-\hat{{VE}_{Delta}(u))}$ and $\hat{\beta_{ve\_delta(u)}}=log(1-\hat{{VE}_{Delta}(l))}$, respectively. We computed a standard error ${SE}_{\beta_{ve\_delta}}=\frac{\hat{\beta_{ve\_delta(u)}}-\hat{\beta_{ve\_delta(l)}}}{2*Z_{0.975}}$, where $Z_{0.975}$ represents a quantile of standard Normal distribution, and generated pseudo-random numbers from Normal distribution:

$\hat{\beta_{ve\_delta}}\sim Normal\left( \hat{\beta_{ve\_delta}} ,{SE}_{\beta_{ve\_delta}} \right).$

Simulated values of $\hat{\beta_{ve\_delta}}$ were transformed back to original scale using following formula: ${VE}_{Delta}=1-exp(\hat{\beta_{ve\_delta}}$).

To construct empirical distribution of ${OR}_{case-case}$we generated pseudo-random numbers from Normal distribution $\hat{\beta_{OR_{case-case}}}\sim Normal\left( \hat{\beta_{ORcase-case}} ,{SE}_{\beta_{ORcase-case}} \right)$ where $\beta_{OR_{case-case}}$ represents coefficient from logistic regression model and ${SE}_{\beta_{ORcase-case}}$ its standard error
To return to original scale, we applied an inverse transformation:

${OR}_{case-case}=exp(\beta_{OR_{case-case}})$.

We draw 10 000 samples of ${OR}_{Delta}$ and ${VE}_{Delta}$used them to construct empirical distributions of ${VE}_{Omicron}$

$${VE}_{Omicron}=1-\left[ \left( 1-{VE}_{Delta} \right)\cdot{OR}_{case-case} \right]$$

The 2.5 and 97.5 percentiles of these empirical distributions were used as lower and upper limits of the 95% confidence intervals for ${VE}_{Omicron}$
